# Impact of nursing robotic assisted surgery education on Kirkpatrick's four levels

**DOI:** 10.3389/fsurg.2025.1638559

**Published:** 2025-08-11

**Authors:** Maria Angela Cerruto, Elisabetta Veronese, Francesca Moretti, Nadia Mansueti, Marianna Azzolini, Erika Poiesi, Nicola Vezzari, Erika Vignola, Davide Gulotta, Andrea Barp, Alessandro Veccia, Antonio Benito Porcaro, Riccardo Giuseppe Bertolo, Alessandro Antonelli, Romina Leardini, Luca Dal Corso, Massimo Zanolli

**Affiliations:** ^1^ Service for Professionalism Development and Innovation, University of Verona, Azienda Ospedaliera Universitaria Integrata, Verona, Italy; ^2^ Healthcare Professions, Azienda Ospedaliera Universitaria Integrata, Verona, Italy; ^3^ Department of Urology, University of Verona, Azienda Ospedaliera Universitaria Integrata, Verona, Italy; ^4^ Healthcare Professions, University of Verona, Azienda Ospedaliera Universitaria Integrata, Verona, Italy

**Keywords:** robotics, nursing, education, Kirkpatrick model, surgery, urology, gynaecology, training

## Abstract

**Introduction:**

As the robotic surgical burden of diseases increases, the development of skilled surgeons and nursing surgery teams is of fundamental importance. The Kirkpatrick Model is a widely recognised evaluation framework that helps educators assess the impact and effectiveness of educational interventions. However, there is little evidence regarding the effectiveness of robotic surgical training programmes for nursing teams. The study aimed to develop an organisational improvement model to identify the profile of a nurse expert in Robotic Assisted Surgery (RAS) and to define a suitable training programme according to Kirkpatrick's evaluation levels. The model also aimed to increase the number of active robotic rooms while maintaining the same number of staff.

**Methods:**

From January 2023 to December 2024, a single hospital institution developed an organisational improvement project consisting of the following steps:- Identification of the catalogue of competences of the nurse expert in RAS, using the contributions of personnel already active in this field;- Mapping of competences;- Start of a training programme, evaluated according to the Kirkpatrick model;- Remapping of competences at the end of the training;- Gradual increase in the number of active robotic operating theatres by 2024.

**Results:**

A total of 58 operating room nurses, including the 15 experienced nurses already on staff, had their competencies mapped. At the end of the training programme, 81% of these nurses had achieved the level of competency required to operate a robotic operating room independently, resulting in an overall increase of 154% in the number of active robotic operating rooms per week.

**Discussion:**

Detailed planning of training activities dedicated to existing operating theatre nurses, eliminating the need for additional recruitment, together with periodic monitoring of newly acquired skills, has enabled the number of active robotic operating theatres to increase, significantly impacting the company's organisational model.

## 1 Introduction

Robotic Assisted Surgery (RAS) is one of the most significant innovations in surgical procedures, offering advantages such as greater precision, minimally invasive techniques and faster recovery times for patients. However, the success of these advanced techniques also hinges on the preparation and expertise of the support team, especially nurses, who are instrumental in ensuring the safety and efficacy of these interventions. Moreover, there is no single benchmark to identify the profile of a nurse expert in RAS or define a suitable training programme. Many studies analysed learning curves in robotics using at least 20 surgical procedures (1). It is therefore possible to argue that perioperative staff, including nurses, benefit from a minimum exposure of about 20 cases to attain a baseline skill level.

For these reasons, adequate and targeted training of nurses in RAS is essential, as it improves technical skills, optimises operating procedures, and ensures high-quality care (2, 3). According to Kirkpatrick's model (4), the effectiveness of this training can be evaluated in terms of not only satisfaction and learning, but also changes in professional behaviour and tangible results for patients.

This ensures that the training investment produces lasting and tangible results, improving clinical practice and safety in the operating theatre, and fostering better care quality, resource management and organisational effectiveness and innovation.

The main objectives of the study were therefore to develop an organisational improvement model to identify the profile of an expert nurse in RAS, implement a training programme for operating room nurses to acquire transversal skills in robotics and increase the number of active robotic rooms while maintaining the same number of staff.

## 2 Materials and methods

An educational training programme was implemented in a single Italian Tertiary Hospital from January 2023 to December 2024 with the aim of improving the RAS skills of operating room nurses. This project involved the following steps: (1) To define the nursing skills profile in RAS (for the Da Vinci, Versius and Hugo platforms) by creating a special catalogue with the help of nurses who are already working in this field. (2) To map the skills of operating theatre personnel. (3) Planning all training activities, including lectures, video viewing on an intranet platform, simulator-based activities in the operating theatre, shadowing experienced personnel, and individual mapping, for both newly recruited nurses specialising in RAS and existing personnel skilled in RAS with the Da Vinci platform, as well as for the management of surgical activities with the Versius and Hugo robotic platforms.

### 2.1 Catalogue of nursing skills in RAS

The Catalogue of Nursing Skills in RAS was defined using 15 nurses already on staff in the robotic operating room. This Catalogue was developed, according to the Association of periOperative Registered Nurses (AORN) (5), as a tool for identifying the activities and processes carried out by dedicated nurses in the context of RAS. This tool outlines the activities to be performed and the skills required by the organisation for this experienced professional role. The catalogue is organised by the stages of patient care within a macro-specialist area, such as urology, gynaecology, gastric surgery or general surgery: pre-operative phase (preparation for the surgical procedure; setting up of the operating room; sterile instrumentation); intra-operative phase (instrumentation during the surgical procedure); post-operative phase (closing of the surgical procedure; rearrangement of the operating room). The following additional relevant elements were also included in the context of the operating block: urgency/emergency conversion, problem management and coping with machine errors. Each specialist macro-area has been assigned a set of behaviours/activities. These indicate the reference standard for an activity “done well”. Table 1 describes all the behaviours to be followed in the Catalogue of skills to be mapped, which are divided by stages of patient care and by relevant elements. In accordance with level 3 of Kirkpatrick's model, each behaviour was given a score from 0 to 5 (0–1: novice; 2: advanced beginner; 3: competent; 4: proficient; 5: expert; according to Dreyfus five-stage model of adult skill acquisition) (6). A minimum score of 3 is required to achieve autonomy for each behaviour. Table 2 describes the definition of each skill acquisition stage and related score.

**Table 1:** Catalogue of nursing skill to map the competencies of the RAS nurse in accordance with the stages of patient care and additional relevant elements.

Stages of patient care and additional relevant elements	Behaviour
Pre-operative	In the event of intraoperative complications (e.g., bleeding), the nurse prepares the surgical instruments for a videolaparoscopy/laparotomy in a sterile field, depending on the type of surgery
	The day before, the nurse's task is to examine the operating lists and plan the use of the necessary materials for all operations that are scheduled
	The operating list is updated by the nurse on the day of surgery, with any necessary changes being made
	The nurse sets up the operating theatre in cooperation with the theatre operator, performing the following tasks:
	- Preparing the robotic cables - Supervising the switching on of the robot - Checking the system according to the robotic software indications - Supervising and checking the switching on of the necessary electro-medical equipment
	In the event of a deviation from the standard procedure, the nurse shares the necessary electro-medical equipment with the colleague in the operating room
	The nurse prepares any materials that may be needed if the surgical procedure deviates from the standard
	The instruments and robotic procedural package are prepared by the nurse for the individual planned surgery
	The nurse prepares the necessary disposables in a sterile field, taking into account the type of surgery
	Nurse shares possible risks with colleagues and the medical team: - Obesity - Bleeding - Previous surgery
	Prepares surgical instruments for laparoscopy/laparotomy in case of intraoperative complications (e.g., bleeding)
Intra-operative	Before the procedure begins, the nurse must inform the surgeon about any critical issues that have been identified
	The nurse suggests possible solutions to the critical issues that have been identified
	Connect the robotic instrument to the correct “energy” cable (green/blue)
	The nurse removes and repositions the robotic instrument on the robot arm
	Introduction of the robotic instrument inside the trocar without pushing it into the cavity
	The nurse prepares and assists with the passage of gutters, suture threads and hemostats, depending on the type of procedure
	The materials used in the cavity are tracked by the nurse, who keeps count of them on the serving table
	The necessary devices at the bleeding site are anticipated by the nurse
	The nurse either performs the preparation in the operating field or supervises the theatre operator performing the preparation, depending on the type of haemostat needed
	Notify the medical team of any critical issues relating to the counting of materials
	The room operator and the nurse work together to count the materials
	During the conversion, the nurse communicates to the room operator the actions to be performed in order of priority
	The nurse sets up the conversion from RAS to laparoscopic or open surgery
	In an emergency situation (e.g., bleeding), the nurse is responsible for organising the conversion from robotic to open procedure
	When converting, the nurse must first identify whether it is possible to perform a material count
Post-operative	The robotic devices are reprocessed by the nurse at the end of surgery, before being sent for sterilisation
	It is the nurse's job to arrange the urgent dispatch of robotic devices to be sterilised, so that they are ready for use in subsequent procedures
	The nurse organises the dispatch of surgical instruments for sterilisation and reports any issues or maintenance required
	The nurse organises the dispatch of the robotic instruments to the sterilisation area, reporting any issues and contacting the relevant person/coordinator directly if necessary
	The nurse rearranges the operating room for the next surgical procedure, ensuring everything is in place and ready to go
Urgency emergency conversion	The nurse prepares the sterile dressing for the first operator at the robotic console in advance
	Alerting the theatre operator to call for help
	The removal of the robotic equipment from the operating field is coordinated by the nurse
	The nurse promptly approaches the instrument cart set up for the conversion and removes all devices that do not allow it to proceed
Troubleshooting	Troubleshoots off-site robotic equipment errors and activation the expert advice when necessary
	Clean the optical fibres before replacement in case of fogging
	Resolution of errors appearing on the “herb” generator
	Activation of the maintenance request if the error is not resolved
Cope with device errors	The nurse handles the “orange light” error (reversible error): - realigns the instrument - moves the instrument's wheels - executes release and re-engagement on the arm
	The nurse handles the “red light” error (reversible error): - disposes of the release key - orders the room operator to restart the equipment

**Table 2:** Description of each skill acquisition stage and related score in the assessment of behaviour competences.

Score	Skill stage	Behaviour competence description
0	Novice	The nurse has no experience of this process/activity
1	Novice	The nurse partially adheres to the expected standard for that process/activity and requires the support of an experienced colleague
2	Advanced Beginner	The nurse adheres to the expected standard for that process or activity, but occasionally requires the support of an experienced colleague
3	Competent	The nurse fully adheres to the expected standard for that process or activity and may also support other colleagues
4	Proficient	The nurse fully adheres to the expected standard and acts as a supervisor for their colleagues
5	Expert	The nurse fully adheres to the expected standard. They act as a supervisor for their colleagues. They are also a reference point for that process/activity

### 2.2 Training program

The training programme consisted of three phases. Phase 1 involved 9 h of theoretical and practical classroom lessons from January to March 2023. Phase 2 involved 12 h of dry lab simulation on robotic platforms (Da Vinci, Versius and Hugo) from April to June 2023. Phase 3 involved field training in the RAS operating theatre alongside experienced personnel from July to December 2023.

The theoretical and practical classroom lectures covered the following topics:

- The management of surgical conversions
- The point of view of the medical examiner and the jurist
- Overview of accessory materials and specialist instruments
- How to prevent patient positioning complications
- Correct mattress positioning
- Patient positioning systems
- Patient temperature management and Trendelenburg
- Overview of set-up preparation in the different surgical specialties
- Emergency management in RAS
- Troubleshooting, simulation and error management
- Scenario simulation and debriefing

The Kirkpatrick model (4) was used to evaluate the effectiveness of nursing education, considering the four levels:

1. **Reaction**: This level measures how nurses perceived the training course using a narrative approach. They were asked if they found the RAS training useful and interesting, and if they felt involved and motivated. In addition, participant satisfaction was measured using an institutional questionnaire containing three questions, scored using a 5-point Likert scale (see below).
2. L**earning**: This level assesses whether nurses have acquired the necessary skills and knowledge to use surgical robots correctly and handle procedures, through practical exercises.
3. **Behaviour:** This level concerns how nurses apply the new skills in the field to achieve an adequate degree of competence.
4. **Results**: The focus of this level is on evaluating the comprehensive effect on the hospital organisation as a whole, with a particular emphasis on the augmentation of active robotic operating theatres.

To assess trainees' reactions, we surveyed their satisfaction with a 5-point Likert global rating scale (1–5) of about 3 items: (1) How do you assess the relevance of the topics covered with respect to the subsequent need for updating?; (2) How do you assess the educational quality of this training programme? (3) How do you rate the usefulness of this training event for your training/upgrading?

To assess learning, a checklist was used in which the trainer indicated whether the learner had achieved the following learning objectives at the end of the first two phases of the training programme:- The learner learnt how to make connections and switch on the system;—The learner learnt how to configure the system according to the different types of surgical procedure;—The learner learnt how to handle and recognise standard and specialised poly-use and single-use instruments;—How to handle the main types of potentially occurring errors;—How to manage emergencies in RAS (conversion);—General rules of patient positioning in RAS.

For the evaluation of behaviour, at the end of phase 3 of the training programme, the competences were remapped as per the catalogue. The behaviour assessment was carried out by two experienced operating room nurses (E.V. and L.D.C.) from the research team. They had a Master's degree in nursing coordination and were appropriately trained in the use of Likert-type scales. The two assessors conducted the evaluations independently, and any disagreements were discussed to reach a consensus on a final, unambiguous assessment.

The final profile of autonomous nurse in RAS was defined as follows:

**Competent:**

- At least 20 robotic surgery cases assessed.
- Completion of the formal robotic surgery training programme.
- Passing the competency assessment with a minimum score of 3 for each behaviour.

**Proficient:**

- At least 30 robotic surgery cases assessed.
- Completion of the formal robotic surgery training programme.
- Passing the competency assessment with a minimum score of 4 for each behaviour.

**Expert:**

- At least 50 robotic surgery cases assessed.
- Completion of the formal robotic surgery training programme.
- Passing the competency assessment with a minimum score of 5 for each behaviour.

Kirpatrick Level 4 was evaluated in terms of the total number of active operating theatres and patients undergoing RAS in 2024 compared to 2023. All surgeons involved are experts in robotics and their numbers did not change during the study period.

### 2.3 Statistical analysis

Descriptive statistical methods were used to identify the basic characteristics and results of the general education survey of the study population. The overall reaction of the participants was expressed in mean and standard deviation according to each item. To assess learning, changes in participants' overall competency and their achievement of goals and objectives of each surgical skill from beginning to end were assessed with a paired t test. Behaviour change was expressed with mean and standard deviation. Spearman's Rank Correlation was used to measure inter-rater reliability (IRR) between assessors. All statistical analyses were performed using SPSS Statistics version 29.

## 3 Results

A total of 58 operating room (OR) nurses, including the 15 identified as expert nurses (both OR and first assistant nurse; 13 females and 2 males), were mapped to construct the competence catalogue. From January to December 2023, all nurses with a non-expert profile (43/58; 9 males and 34 females; mean score 1.22 ± 1.22) undertook the training programme, receiving theoretical and practical training in a dry lab (phases 1 and 2) and in the field (phase 3).

### 3.1 Level 1: reaction of trainees

At the end of the phase 1 and 2 training programme, a 5-point global rating scale survey was conducted among the 43 trainees. The overall satisfaction score was 4.79 ± 0.42 out of 5.00, which is between “good” and “very good”. Trainees rated the programme highly in terms of its relevance to their needs and interests (4.86 out of 5.00), its educational quality (4.72 out of 5.00) and its usefulness for personal development (4.81 out of 5.00). Only one trainee found the programme useful. None of the trainees thought that the training event was poor or neutral. Using a narrative approach, the following feedback was left by most of those who had not had previous experience of robotics: “The course was well structured, but some of the theoretical notions could be expanded upon in more surgical areas than the room. For those who have never used the robot or use it infrequently, it would be useful to have an introductory section on all the robotic components, the irons and the sterile dressing of the robot, as well as their specific names. The simulations that help you realise the difference between theory and practice were also very useful.”

### 3.2 Level 2: learning

All participants had achieved all the required learning objectives by the end of phases 1 and 2 of the programme.

### 3.3 Level 3: behaviour change

**Figure 1:**
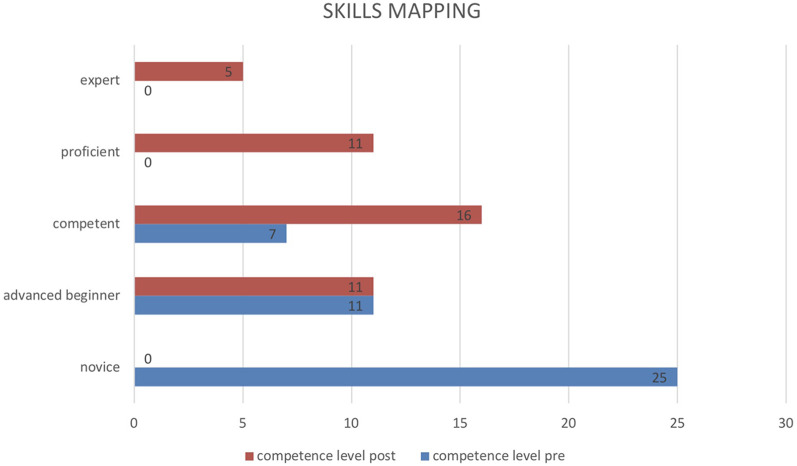
Distribution of competence levels before and after completing the full training programme.

By the end of the entire training programme, 32 out of 43 nurses (mean score: 3.45 ± 0.95, *p* = 0.000) had demonstrated the ability to independently manage a robotic operating theatre from a nursing perspective (at a competent level). IRR between assessors was significantly high (0.992; *p* > 0.01). Figure 1 shows the distribution of competence levels before and after completing the training programme.

### 3.4 Level 4: organisational change results

**Figure 2:**
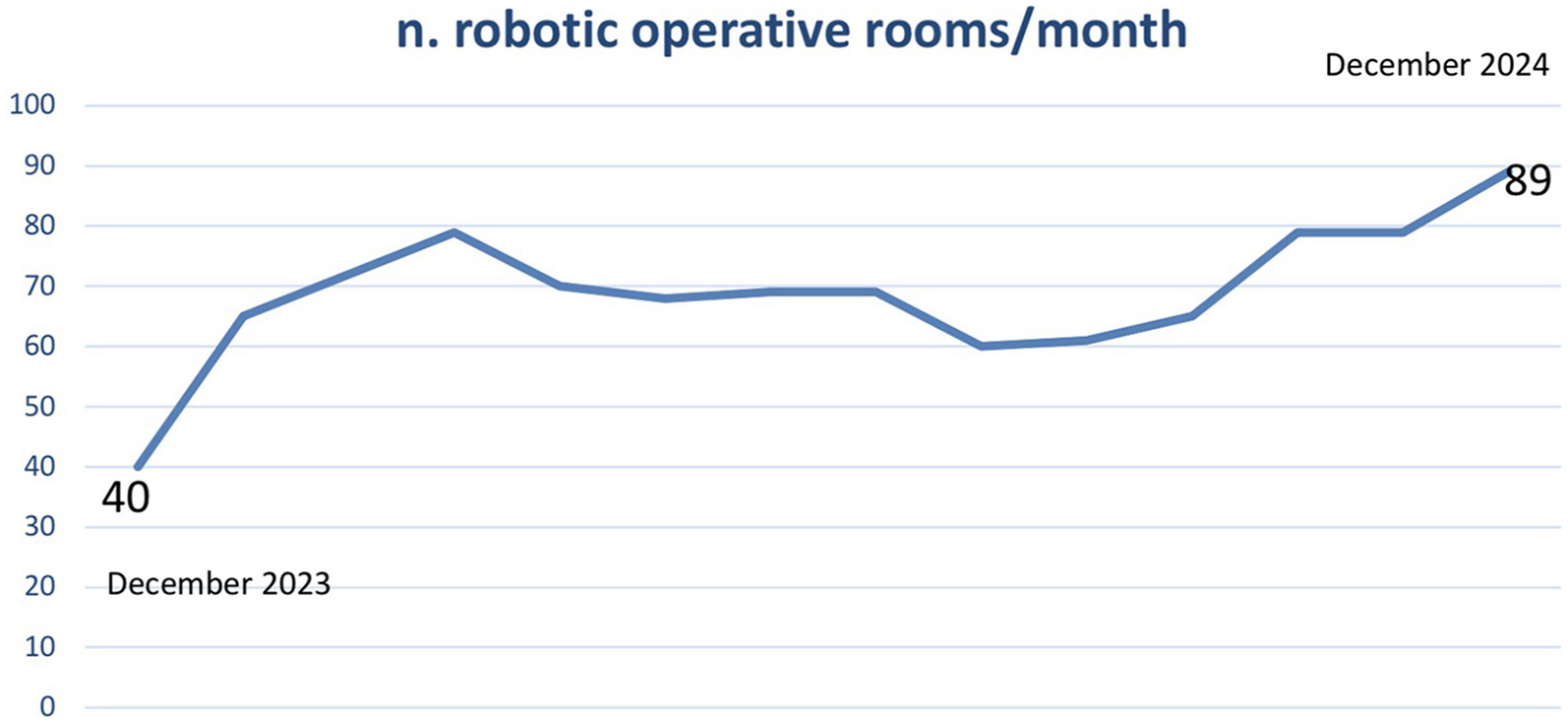
Trend in the monthly number of robotic operating theatres activated from December 2023 to December 2024.

**Figure 3:**
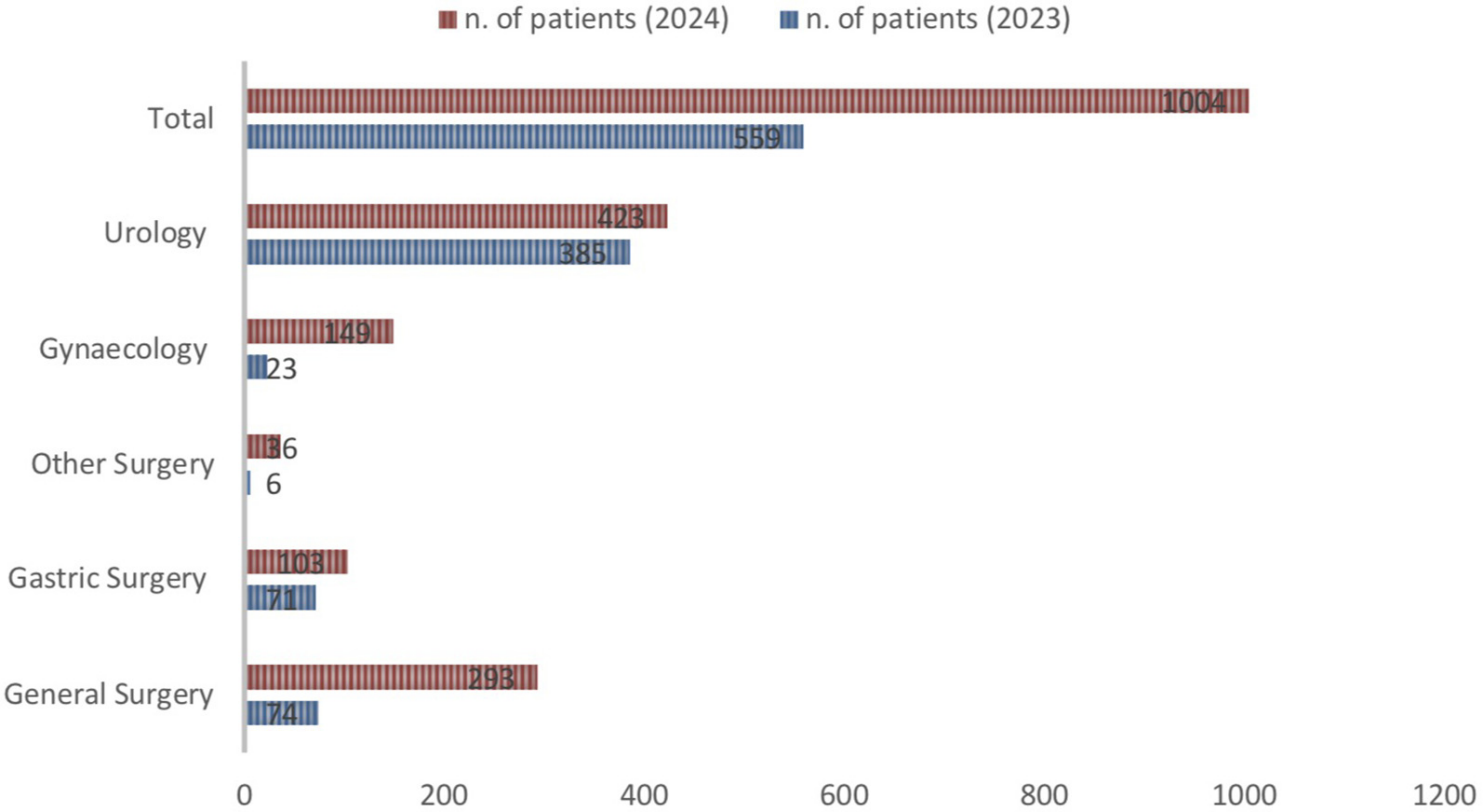
Number of patients treated with robotic stratified by type of surgery, 2023 vs. 2024.

A total of 859 robotic operating theatres were activated in 2024 vs. 480 in 2023, with an overall increase of 154% (Figure 2). The number of operating theatres that were activated increased in all of the macro-specialty areas identified (Urology 384 in 2024 vs. 288 in 2023; Gynaecology 203 vs. 0; Gastric surgery 127 vs. 96; General surgery 145 vs. 96). This resulted in an increase in the total number of patients treated with RAS in the (559 in 2023 vs. 1004 in 2024) (Figure 3).

## 4 Discussion

Kirkpatrick's four-level model of evaluation and training assesses participants' reactions to the training, what they learn from the programme, how they apply the knowledge in real practice and transfer it to others, and the results of the training programmes with regard to clinical outcomes and organisational goals and objectives (4). This model is widely used to evaluate nursing education. However, the outcomes level (level IV) is the most challenging to achieve (7). The strength of our study is that, to our knowledge, it is the first to meet all four levels of the Kirkpatrick model in robotic nursing training. Indeed, few studies in the literature have directly investigated Kirkpatrick levels in the context of robotic nurse training. Instead, they tend to address related issues, such as the effectiveness of robotic surgery training and its potential impact on nurses' skills and clinical outcomes.

As far as Level I is concerned, most of the participants in our study were satisfied with the training programme they underwent. The feedback from those with no prior experience of RAS is consistent with findings in the literature. Indeed, a recent survey of 114 operating room nurses working in 12 institutions that have performed robotic surgery in Turkey demonstrated that nurses have a favourable opinion of robotic surgery; nurses with experience in robotic surgery exhibit higher levels of individual innovativeness; and nurses who have received training are significantly more adapted (8). A recent literature review on education and training of operating room nurses in RAS found that only 3 studies described the development of training programmes for operating room nurses working with RAS (2, 9, 10, 18). In 2001, Connor et al. described a learning laboratory for OR nurses working with a cardiothoracic surgical robot. They concluded that the approach to adapting this kind of technology should be stepwise and parallel to its development (9). A RAS programme to educate and assess OR nurses' thoracic RAS competencies for both technical and non-technical skills was described by Sarmanian et al. They described how an OR equipped with robots would be ideal for learning exercises, as learners could immerse themselves in the actual practice environment. They also emphasised the importance of leadership support for success (18). None of them focused on Levels 1 or 2 of the Kirkpatrick model.

A study evaluated medical and nursing students' knowledge of and interest in robotic surgery, analysing how this may change after a hands-on training course using new Hugo RAS and Versius System simulators (11). Before the hands-on course, 44.0% of nursing students were interested in surgical disciplines and robotic surgery. After the simulation, all students reported a high level of interest in robotic surgery and some required a dedicated internship (*p* < 0.001). The students also provided feedback on the ease of use of the robotic simulators (on a scale 0–10), with a median score of 8, and no differences between nursing and medical students. This study demonstrated a great interest in robotic surgery as part of medical and nursing education.

We observed that at the end of the training programme, 32 out of 43 nurses (with a mean score of 3.45 ± 0.95, *p* = 0.000) proved able to manage a robotic operating theatre independently from a nursing point of view. Collins et al. developed a new, stepwise robotic training programme to broaden and standardise training for bedside assistants (19). Thirteen participants were evaluated to determine if the training programme enhanced their confidence and comfort. A survey was conducted before and after the training programme and at different stages. All participants reported statistically significant increases in confidence and comfort after the training programme (*p* < 0.001) and at each stage (*p* < 0.001). Feedback regarding curriculum improvement was obtained, suggesting more training and practice in smaller groups. All participants felt RAST was beneficial and that it should be implemented as standard training during onboarding for all robotic bedside assistants.

Using Likert-type scales to assess behaviour may expose several limitations, such as the tendency to give a central response, subjective interpretation of options, the assumption of equal intervals and limited qualitative depth (12). Nevertheless, these scales are often used in literature, particularly in training, due to certain strengths (13, 14). For example, they can be used to evaluate attitudes and perceptions (15). In training, it is crucial to understand how involved, motivated or satisfied participants feel, and the Likert scale is perfect for this. They can also be used to measure perceived effectiveness. Participants can be asked to express their degree of agreement with statements. The Likert scale offers a common metric, making it easy to standardise and compare results. It is also easy to administer, as Likert-type scales work well both on paper and in digital format, and data interpretation analysis is straightforward.

Level IV of the Kirkpatrick model identifies the added value of a programme to society by evaluating intervention design application in health professionals' practice and the results of its use in a local context (16). The most common reason for excluding Level IV was the need for medium- and long-term follow-up periods, as well as the complexity of relating outcomes to the training programme and distinguishing them from other factors (17). Kirkpatrick's level IV is considered the most challenging as it assesses the impact of training on organisational practice. This requires additional instruments, information and stakeholder input, as well as more time. We overcame these issues by promoting the project to the hospital's strategic management. This enabled us to manage and coordinate scheduling, space, training and human resources, achieving the objectives for organisational change. There are no data in the literature for comparison.

The number of active robotic operating theatres has increased significantly as a result of a combination of detailed planning of training activities for existing operating theatre nurses, which has eliminated the need for additional recruitment, and periodic monitoring of newly acquired skills. This has had a substantial impact on the company's organisational model. During the study period the number of procedures per room per day did not increase. To assess the impact of our training programme also on the number of procedures per operating room per day, a longer follow-up period is required. Monitoring this parameter in future could certainly provide further data on procedure productivity and workflow efficiency, as well as information on the increase in the system's overall capacity (18).

## Data Availability

The raw data supporting the conclusions of this article will be made available by the authors, without undue reservation.
